# Multi-Stage Pedestrian Positioning Using Filtered WiFi Scanner Data in an Urban Road Environment

**DOI:** 10.3390/s20113259

**Published:** 2020-06-08

**Authors:** Zilin Huang, Lunhui Xu, Yongjie Lin

**Affiliations:** School of Civil Engineering and Transportation, South China University of Technology, No.381, Wushan Road, Guangzhou 510641, China; ctjulian@mail.scut.edu.cn (Z.H.); lhxu@scut.edu.cn (L.X.)

**Keywords:** pedestrian positioning, WiFi detector, distance estimation, piecewise polynomial regression, Taylor series

## Abstract

Since widespread applications of wireless sensors networks, low-speed traffic positioning based on the received signal strength indicator (RSSI) from personal devices with WiFi broadcasts has attracted considerable attention. This study presents a new range-based localization method for outdoor pedestrian positioning by using the combination of offline RSSI distance estimation and real-time continuous position fitting, which can achieve high-position accuracy in the urban road environment. At the offline stage, the piecewise polynomial regression model (PPRM) is proposed to formulate the Euclidean distance between the targets and WiFi scanners by replacing the common propagation model (PM). The online stage includes three procedures. Firstly, a constant velocity Kalman filter (CVKF) is developed to smooth the real-time RSSI time series and estimate the target-detector distance. Then, a least squares Taylor series expansion (LS-TSE) is developed to calculate the actual 2-dimensional coordinate with the replacement of existing trilateral localization. Thirdly, a trajectory-based technique of the unscented Kalman filter (UKF) is introduced to smooth estimated positioning points. In tests that used field scenarios from Guangzhou, China, the experiments demonstrate that the combined CVKF and PPRM can achieve the highly accurate distance estimator of <1.98 m error with the probability of 90% or larger, which outperforms the existing propagation model. In addition, the online method can achieve average positioning error of 1.67 m with the much better than classical methods.

## 1. Introduction

Outdoor pedestrian positioning in the urban road environment is a challenging but significant topic in the field of intelligent transportation systems (ITS), which has great application prospects, such as transportation planning, security monitoring, pedestrian counting, traffic signal optimization and traffic guidance. Various moving sensors have been developed based on passive and active positioning technologies for capturing mobile target movement dynamics [1]. However, due to complicated factors like the unknown target moving and its outdoor surrounding environment, it is difficult to locate and track mass of mobile targets in cluttered urban road environments, such as vehicles, pedestrians, and cyclists.

Generally, the Global Positioning System (GPS) is considered to be a dominant technology for outdoor localization because of its worldwide availability and high positioning accuracy. However, it requires users to install and operate a mobile application to spontaneously transmit GPS data to the remoted center, which is inconvenient and a serious challenge to battery-based user devices in real life. Meanwhile, GPS-based approaches are also unreliable or even unavailable in dense urban areas owing to the signal attenuation or blocking caused by skyscrapers, tunnels, underground space, and other construction materials [2].

Many researchers have proposed a series of alternative localization schemes to address this issue, including ultrasonic-based [3,4], radio-frequency (RF)-based [5,6], ultra-wideband (UWB)-based [7], and video camera-based [8] schemes. Most systems are usually operated at the cost of masses of users and easily affected by weather conditions, which cannot be used for large-scale individual localization on the citywide road network. With the popularization of smartphones and their improvement, integrated sensors into private terminals have become increasingly abundant, which makes it possible to provide location-based service (LBS) to users at anytime and anywhere. In mainland China, for instance, the adoption rate of mobile phones has reached up to 94.5%, and about 85.8% of netizens are connected to the Internet service via their advanced mobile devices [9].

Nowadays, most smartphone-based localization technologies commonly focus on cellular networks [10], Bluetooth [11], and WiFi [12,13,14,15]. Among, cellular network-based approaches have been suggested as effective ways to track cellphones based on their cellular signal strength. However, the location estimation is very coarse and only appropriate for traffic origin-destination (O-D) surveying [16]. Besides, the penetration rate of Bluetooth in smartphones is usually between 5% and 12%, because most applications involving Bluetooth technology are carried out in the vehicle network [17]. In contrast, WiFi sensing is able to transmit more information when it tries to connect to surrounding access points (APs), which could increase the chances of being detected. Furthermore, this method does not need extra hardware or software for users and takes up fewer social resources except only deploying WiFi detectors along the urban road network [18,19].

The key information in WiFi-based localization scheme is the received signal strength indicator (RSSI), which can be easily obtained from WiFi-enabled private devices (e.g., smartphone, iPad, notebook computer, smart watch, connected vehicle) [20,21,22,23,24]. As a result, the pervasively available WiFi infrastructures appear to be a promising choice for city planners and traffic engineers to estimate traveler origin-transfer-destination (OTD) movements and flows, which are not collected by existing vehicle-oriented sensors (loop detector, magnetic detector, microwave, radar, video, etc.).

By contrast with cellular network and GPS-based localization technologies, most existing WiFi-based approaches are mainly considered to be used in the indoor environment. Unfortunately, WiFi-based indoor localization methods cannot be simply transferred into urban road environments, so there has not been satisfactory localization accuracy so far, whether from academic proposals (e.g., [14,15]) or industrial practices (e.g., [25,26]). Technically, most academic proposals are trying to migrate the existing WiFi fingerprinting methods for indoor localization into urban road environments (e.g., [14]), but it needs prior knowledge of the deployment site and large-scale repetition experiments for acquiring a stable finger printing database for each divided map tile. However, under the large-scale outdoor scenarios, it is extremely difficult and expensive to locate citywide thousands or even millions of road links using WiFi fingerprinting methods.

To further explore the application of WiFi-based outdoor localization with high accuracy and robustness, this study analyzes the positioning principle and technical architecture based on RSSI, and presents an innovative two-stage method for low-speed pedestrian positioning. In the offline phase, we propose to apply the piecewise polynomial regression model (PPRM) technique to establish the RSSI-distance formula. In the online phase, this study proposes a combination of constant velocity Kalman filter (CVKF) and PPRM to filter the real-time RSSI value and estimate the Euclidean distance between the target and WiFi detectors. Subsequently, the least squares Taylor series expansion (LS-TSE) algorithm is employed to estimate the initial target location, and the unscented Kalman filter (UKF) is used to smooth the final target location according to the series of historical estimated positioning points. Our field experiments show that the proposed method has good localization accuracy.

In short, the contributions of this paper can be summarized as follows:

- To solve the problem that it is difficult to accurately establish the RSSI-distance relationship in the RSSI-based positioning scheme, we propose a novel PPRM to capture the uncertainty of RSSI fluctuation. By contrast with the traditional propagation model (PM) or polynomial regression model (PRM), the developed RSSI–distance relationship can be formulated into a dynamic *n*th-degree polynomial to improve Euclidean distance estimation for pedestrian localization.
- Different from the previous filtering algorithms for indoor environment, we propose a new CVKF fusion algorithm to handle real-time RSSI fluctuations for the outdoor pedestrian positioning, and prove that CVKF + PPRM can further improve the distance estimation accuracy.
- We design an entire system of a multi-stage pedestrian positioning by using the combination of PPRM, CVKF, LS-TSE and UKF, which can achieve high-position accuracy performance in an urban road environment. By contrast with the GPS-based method that requires users to install software and initiate positioning requests, this positioning scheme based on WiFi scanner data can real-time locate pedestrians to help transportation agencies better monitor the abnormal situation of pedestrian flow and behavior.

The remainder of this paper is organized as follows. Section 2 briefly reviews the related work. The detailed methodology is formulated in Section 3. Section 4 describes field experiments and analyzes the localization results. Finally, conclusions and future work are drawn in Section 5.

## 2. Related Work

The use of WiFi positioning technologies has been widely discussed by many researchers in the past decade, and the most commonly adopted localization prototype is to use RSSI [14,15]. In terms of whether distance estimation is required or not, schemes can be divided into two groups: range-free and range-based methods [15].

The range-free localization methods do not need to utilize the physical distance to determine terminal location. One of the most widely used range-free methods is fingerprinting localization, which mainly investigates the difference between the received mobile device’s fingerprint from multiple WiFi scanners and the reference points (PRs) [27] or the occurrence probability at the same location [28]. The performance of these methods depends on the quantity of reference points adopted per unit area, namely, the reference point density. In order to relieve the training burden while maintaining the performance, many scholars have proposed various improvement measures. Liu et al. [29] have proposed a transfer learning-based framework to enhance the scalability of the fingerprint-based indoor localization framework by reducing offline training cost without affecting the accuracy of localization. Sun et al. [30] have proposed a Gaussian process regression model to predict the fingerprint spatial distribution of signal strength at the uncalibrated area to amplify fingerprints when the reference points are limited. Kuo et al. [31] enriched the reference points by interpolating some virtual points and creating their fingerprints. Generally, these measurements might help partly to reduce the fingerprint calibration efforts, but will fail on the large-scale urban road network for travelers’ localization.

By contrast, the range-based methods include RSSI signal filtering and physical distance estimation from WiFi sensors to targets, namely sensor-target distance. In practice, the collected RSSI signals from user devices are very inaccurate and unstable due to noise disturbance at the outdoor environment. Thus, some filtering algorithms have been reported to cope with these noises, such as Kalman filter [32], Bayesian filter [33], and Particle filter [34]. Meanwhile, it is also difficult to accurately calculate the non-linear relationship between RSSI and sensor-target distance due to the cluttered urban road propagation environments. One of the typical estimations is the radio propagation model based on log-distance path loss function, which uses logarithms to describe the RSSI-distance relationship [35,36]. However, these kinds of method are very sensitive to the surrounding environments. Therefore, some researchers have reported improved methods, such as the general regression neural network (GRNN) [32], polynomial regression [11], curve fittings [35] and segmentation fitting method [37]. As known, the main disadvantage of these machine-learning methods is that they generally take multiple iterations to converge to the expected solution based on the huge training dataset. In [38], they conducted a comparative evaluation analysis on four models (log-distance path loss model, exponential model, power model, and polynomial regression model), and found that the polynomial regression held the best results.

In this paper, our proposed two-stage method can achieve a high-accuracy positioning for low-speed pedestrian due to the use of fitted RSSI-distance function by considering the non-linear signal-distance path-loss and continuous target movement tracks along urban roads.

## 3. Model Development

The range-based localization scheme generally can be categorized into two stages: the offline stage and online stage. At the offline stage, the RSSI-distance relationship will be formulated and calibrated, whereas at the online one, the real-time RSSI data of a mobile device is collected and processed for localization. In this section, we present the details of the proposed RSSI-based localization scheme in the urban road environment. For simplicity, this paper only considers the localization in 2-D space and positioning of one moving target (a person), and let *L_t_* = (*x_t_*, *y_t_*) denotes the mobile target location at time *t*.

### 3.1. System Overview

Suppose that there are *M* WiFi detectors and *W* mobile targets corresponding to *W* media access control (MAC) addresses at the road environment. Let *L_m_* = (*x_m_*, *y_m_*) denotes the location coordination of the *m*th WiFi detector, *m*=1, 2, …, *M*. For each WiFi detector, the algorithm searches the same MAC address in the adjacent detector set. Notably, one can easily extract the series of its data via multiple adjacent WiFi detectors, including RSSI, timestamp, the MAC identification of the related WiFi detector. For the *w*th MAC address detected by detector *m*, the RSSI dataset sorted by timestamp is assumed to be $s_{t}^{MW}$. Among, let $s_{t}^{MW}=\{s_{t}^{mw}|m=1,\ldots ,M,w=1,\ldots ,W\}$ denotes the RSSI measurement value of the *m*th WiFi detector for *w*th MAC address at time *t*. The objective of the RSSI-based localization scheme is to estimate the mobile target position on the road network according to all RSSI measurement vectors $s_{0,\ldots ,t}^{MW}=\{s_{0,\ldots ,t}^{mw}|m=1,\ldots ,M,w=1,\ldots ,W\}$.

**Figure 1:**
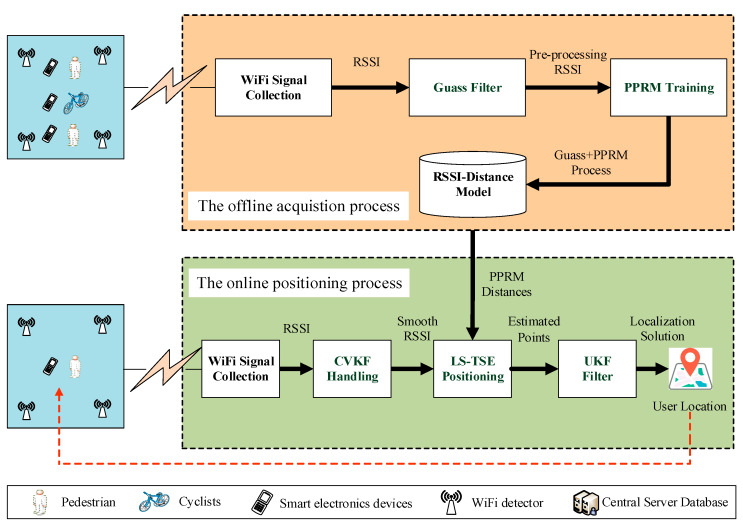
The proposed localization system framework.

The proposed range-based localization scheme is illustrated in Figure 1, which mainly consists of four steps belonging to two stages. The first step is the offline training stage to formulate the RSSI-distance relationship by using PPRM to realize the automatic fitting function segmentation. Also, the specific *n*th-degree polynomial will be calibrated at different fitting function segmentations, which combines a Gauss filter and piecewise function in the training process in order to capture the RSSI propagation characteristics at the urban environment as authentic as possible. The last three steps belong to the online positioning stage. At the second step, the combination of CVKF and PPRM is developed to filter the real-time RSSI value and estimate the sensor-target distance via the estimated RSSI-distance mapping at the first step. The third is real-time to calculate the user’s position by the algorithm of LS-TSE. Finally, this study will smooth the position estimator via UKF by the usage of historical points. Notably, the function of pre-processing Gaussian filtering and the CVKF algorithm in Figure 1 are different. The former is performed during the offline training phase in order to reduce the fluctuation of RSSI value at the stationary observation point. The latter is designed during the online positioning phase such as to reduce the fluctuation of the captured real-time RSSI value.

### 3.2. Received Signal Strength Indicator (RSSI)-Distance Estimation Based on Offline Data

#### 3.2.1. Existing Propagation Model

The common propagation model (PM) for describing the relationship of RSSI and sensor-target distance is the log-distance path loss [35,39]. By using some signal samplings obtained at several observed points, the PM coefficients can be calibrated. Given an observed RSSI, the sensor-target distance can be estimated based on the calibrated PM as follows [35]:

(1) $$PL(d)=PL_{d_{0}}-10\gamma \log\frac{d}{d_{0}}+N(0,\sigma^{2})$$

where, *γ* represents a path-loss parameter related to the specific wireless transmission environment; $PL_{d_{0}}$ is the RSSI at the reference distance *d*_0_; *N*(0, *σ*^2^) is a random variable belonging to zero-mean Gaussian distribution with a variance *σ*^2^; and *PL*(*d*) denotes the detected RSSI corresponding to the transmission distance *d*. Generally, PM with an ideal propagation might work well in line-of-sight (LOS) scenarios in the outdoor spacious environment. However, this model is too simple to obtain accurate distance in cluttered urban road environment due to signal reflection, shadowing and multipath transition from skyscrapers, tunnels, vehicles, concrete walls and other construction materials.

Recently, some researchers have reported that the polynomial regression model (PRM) has a good ability to describe the RSSI-distance relationship [38]. Unfortunately, the attenuation speed of RSSI strength will change as the transmission distance exceeds a certain threshold. Consequently, the goodness-of-fit of the PRM will gradually decline, and the tiny deviation of RSSI can lead to much larger error in distance computation [37]. To overcome this problem, this paper proposes the PPRM to formulate the relationship between RSSI and distance for outdoor localization.

#### 3.2.2. Piecewise Polynomial Regression Model (PPRM)

By contrast with the existing PM and PRM, the proposed PPRM assumes that the RSSI-distance relationship can be formulated into a different *n*th-degree polynomial at the different level of RSSI, and the polynomial coefficients are calibrated by using a training dataset. Moreover, PPRM could automatically divide RSSI into different level to calibrate a series of polynomial regression functions. Correspondently, this study assumes that there are *K* different piecewise segmentations, and let $\Phi_{k}^{w}(s)$ denotes the fitting function at the *k*th segmentations from the *w*th mobile device. In order to simplify model description, we will take a detector and a target to formulate PPRM model as an illustration. Let *s_j_* denotes the RSSI of the *j*th observed point after Gaussian filtering. In this paper, we use a group of linearly independent polynomial functions to build the fitting function:

(2) $$\hat{d}=\Phi (s)={\sum_{k=1}^{K}\left[\lambda (\sum_{i=0}^{n}a_{i}\cdot s_{j}^{i})\right]}_{k},\;i<n\;\mathrm{and}\;k<K$$

where, Φ(s) is the estimated distance from the target to a given WiFi detector; *n* denotes the fitting degree; and *a_i_* represents the coefficient of the *n*th-degree polynomial fitting function, where *i* = 0, 1, …, *n*. The polynomial coefficients can be estimated based on the training dataset. Moreover, if *s_j_* belongs to the *k*th segmentations, *λ* = 1; otherwise, *λ* = 0.

The detailed procedure of the PPRM method in this study is developed as follows:

**Step 1:** Pre-process the RSSI value via Gaussian filter.

Generally, the collected RSSI raw data sharply fluctuates because of the randomness of Radio Frequency (RF) signals. Even at the same location, RSSI will also fluctuate up and down quickly from 0 to 10 dBm [40]. However, numerous reduplicated experiments showed that the distribution of RSSI at a certain point can be seen as a Gaussian distribution [37].

During the offline training phase, we assume that there are *P* raw data at the *j*th observed point from the same sensor. For the *p*th raw data (*p* = 1, 2, …, *P*), the probability density function (PDF) is computed by

(3) $$f(r_{j}^{p})=\frac{1}{\sigma \sqrt{2\pi }}e^{-(\frac{{\left(r_{j}^{p}-\mu \right)}^{2}}{2\sigma^{2}})}$$

where, *μ* and *σ*^2^ denote the mean and variance of RSSI, respectively; $r_{j}^{p}$ means the *p*th raw RSSI data at the *j*th observed point. Generally, the Gaussian filter threshold is set to 0.6 [37]. Thus, the RSSI of the *j*th observed point after Gaussian filtering can be expressed as follows [37]:

(4) $$s_{j}=\frac{1}{P}\sum_{p=1}^{p}r_{j}^{p},\;s.t.\;0.15\sigma +\mu \leq r_{j}^{p}\leq 3.09\sigma +\mu$$

**Step 2:** Solve the *n*th-degree polynomial fitting problem based on the least square’s principle.

The summation of the model fitting square errors is computed by:

(5) $$E(a_{0},a_{1},\ldots ,a_{n})=\sum_{j=1}^{J}{\left[d_{j}-\sum_{i=0}^{n}a_{i}\cdot s_{j}^{i}\right]}^{2}$$

where, *J* is the number of the observed points; *d_j_* represents the real distance from the *j*th observed point to the WiFi detector, *j* = 1, 2, …, *J*; and *s_j_* is the RSSI value at the *j*th observed point. Among, *E* is a quadratic function about the coefficient *a_i_*, and we can achieve the feasible solution of *a_i_* by the least square’s principle. In detail, we set the partial derivatives of *E*(*a*_0_, *a*_1_, …, *a_n_*) to zero with respect to each coefficient *a_i_* as follows:

(6) $$\frac{1}{2}\frac{\partial E}{\partial a_{i}}=\sum_{j=1}^{J}s_{j}^{i}\left[d_{j}-\sum_{i=0}^{n}a_{i}\cdot s_{j}^{i}\right]=0$$

Then, Equation (6) can be converted into the matrix format by:

**AB** = **C** (7)

where, $A=\left(\begin{array}{ccc} \sum_{j=1}^{J}s_{j}^{0}s_{j}^{0} & \ldots & \sum_{j=1}^{J}s_{j}^{0}s_{j}^{n}\\ \vdots & \ddots & \vdots \\ \sum_{j=1}^{J}s_{j}^{n}s_{j}^{0} & \cdots & \sum_{j=1}^{J}s_{j}^{n}s_{j}^{n} \end{array}\right)$, $B=\left[\begin{array}{c} \;a_{0}\\ \;a_{1}\\ \;\vdots \\ a_{n-1}\\ \;a_{n} \end{array}\right]$, $C=\left[\begin{array}{c} \sum_{j=1}^{J}s_{j}^{0}d_{j}\\ \vdots \\ \sum_{j=1}^{J}s_{j}^{n}d_{j} \end{array}\right]$.

The polynomial fitting coefficients $B={\left[a_{0}\;a_{1}\;\ldots \;a_{n}\right]}^{T}$ can be rewritten as:

(8) $$B=A^{-1}C$$

The traditional way to determine the optimal fitting function is to choose the *n*th-degree polynomial with the least mean error (ME) [11]. Although we can search for the fitting function with the least ME, the fluctuation of residual is still relatively large. Therefore, this study employs the sum of the square fluctuation errors (SSFE) as the convergence criterion based on the [41]. For the *n*th-degree polynomial, the SSFE can be calculated as follows:

(9) $$H^{2}=\frac{1}{J-1}\sum_{j=1}^{J}{\left[\sqrt{{\left({\hat{d}}_{j}-d_{j}\right)}^{2}}-\sqrt{{\left({\hat{d}}_{1}-d_{1}\right)}^{2}}\right]}^{2}$$

where, *H*^2^ is the SSFE of *n*th-degree polynomial; ${\hat{d}}_{j}$ denotes the distance estimation from the *j*th observed point to the detector; *d*_1_ means the real value of the first observed point; and ${\hat{d}}_{1}$ represents the distance estimator of the first observed point. Then, the *n*th-degree polynomial with the least SSFE will be selected as the optimal polynomial function.

**Step 3:** Piecewise polynomial fitting segmentation.

The error of each estimated point *F_j_* and the mean error $\bar{F}$ are defined as:

(10) $$F_{j}=\sqrt{{\left(\hat{d_{j}}-d_{j}\right)}^{2}}$$

(11) $$\bar{F}=\frac{1}{J}\sum_{j=1}^{J}F_{j}$$

If the errors *F_j_*, *F_j_*_+1_, and *F_j_*_+2_ of three consecutive points are greater than the mean error $\bar{F}$, the new piecewise function start from *j*th observed point and turn to Step 4; otherwise, turn to Step 5. Therefore, the first data for *k*+1th segmentation fitting function is set to equal to *s_j_*

(12) $$s_{1,k+1}=s_{j},\;s.t.\;F_{j}>\bar{F}\;\mathrm{and}\;F_{j+1}>\bar{F}\;\mathrm{and}\;F_{j+2}>\bar{F}$$

**Step 4:** Re-calibrate the optimal *n*th-degree polynomial fitting function for the new segmentation.

Make a new piecewise function from the point *s_j_* to the last observed point. Re-calibrate the optimal *n*th-degree polynomial fitting function for the new segmentation based on Step 2, and then repeat Step 3.

**Step 5:** Merge all optimal fitting functions.

According to the segmentations obtained by the previous four steps, a series of the optimal fitting functions are merged into a piecewise polynomial fitting model.

**Figure 2:**
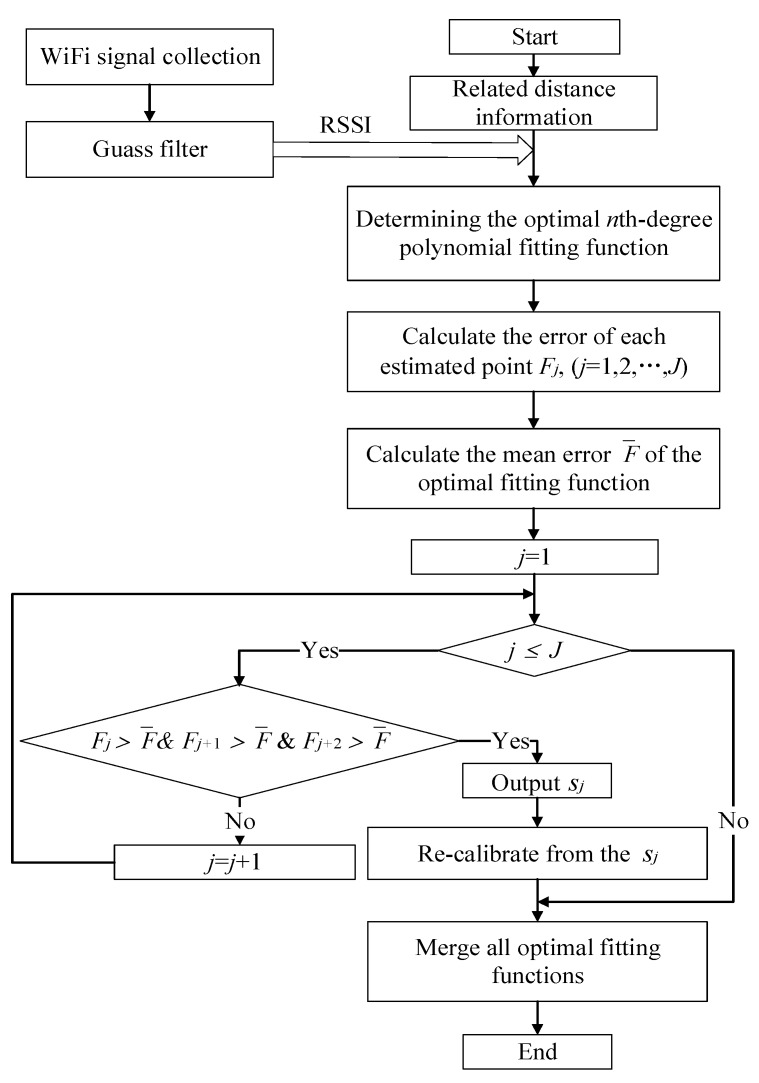
Flow chart of piecewise polynomial regression model (PPRM) algorithm.

The complete procedure of PPRM is given in the Figure 2.

### 3.3. Target Positioning Based on Real-Time Data

At the online positioning stage, this study proposes a combination of CVKF and PPRM to filter the real-time RSSI value and estimate the Euclidean distance between the target and WiFi detector. In addition, we use UKF to filter the estimated positioning points, which can be obtained by LS-TSE. The UKF output are regarded as the final target position.

#### 3.3.1. Real-Time Data Filtering Based on Constant Velocity Kalman Filter (CVKF)

Actually, there is a significant fluctuation in the RSSI value collected by the WiFi detector, and it cannot be directly used for positioning. Kalman filter [32] is a useful algorithm for signal processing to remove the superimposed noise. However, if the error of the first several observed RSSI values is very large, it will have a serious impact on the position accuracy. Therefore, the RSSI value must be pre-processed before using the Kalman filter. A variety of mobility models have previously been described in the literature such as constant-velocity, constant-acceleration, singer acceleration model, mean-adaptive acceleration model [42]. In the previous research work [40], we also proposed a constant velocity Kalman filtering fusion algorithm for noise reduction. In this study, we will employ the constant velocity algorithm to smooth the real-time RSSI. The estimation and prediction stages can be formulated in the following expressions:

(13) $$s_{est(t)}=s_{pred(t)}+\alpha (s_{prev(t)}-s_{pred(t)})$$

(14) $$V_{est(t)}=V_{pred(t)}+\frac{\beta }{T_{S}}(s_{prev(t)}-s_{pred(t)})$$

(15) $$s_{pred(t+1)}=s_{est(t)}+V_{est(t)}T_{S}$$

(16) $$V_{pred(t+1)}=V_{est(t)}$$

where, *s_prev_*_(*t*)_ represents the real-time RSSI measured value at interval *t* from the module of WiFi signal collection in Figure 1; *s_pred_*_(*t*)_ is the predicted value; *s_est_*_(*t*)_ is the smoothed value; *V_est_*_(*t*)_ is the smoothed range rate of the RSSI; *V_pred_*_(*t*)_ means the predicted range rate of the RSSI; *α* and *β* are the gain constants, respectively; and *T_S_* denotes the duration of updated time interval. The smaller the variable α is, the higher the confidence of the predicted value *s_pred_*_(*t*)_ will be, which means the measured error of RSSI is very small. If *β* is too large, the filtering response will be slow because the confidence in the newly measured value is reduced.

Then, the RSSI sequence smoothed by constant velocity algorithm is input into Kalman filter to obtain the final smooth RSSI series [32]. Next, we can substitute the series into the calibrated PPRM model achieve more accurate distance estimation from the moving target to WiFi detectors as shown in Figure 1.

#### 3.3.2. Collaborative Positioning Based on Least Squares Taylor Series Expansion (LS-TSE)

According to the distance from the target to several adjacent WiFi detectors estimated by PPRM at the online phase, we can calculate the target location. In fact, it is a collaborative positioning problem with multiple WiFi detectors. Thus, this study proposes LS-TSE to achieve it. Firstly, assume the coordinate of the target location is (*x*, *y*) and the coordinates of the WiFi detectors are set to (*x*_1_, *y*_1_), (*x*_2_, *y*_2_), …, (*x_m_*, *y_m_*), and then the following equations can be obtained:

(17) $$\begin{array}{cc} {\left(x-x_{1}\right)}^{2} & +{\left(y-y_{1}\right)}^{2}=z_{1}{}^{2}\\ {\left(x-x_{2}\right)}^{2} & +{\left(y-y_{2}\right)}^{2}=z_{2}{}^{2}\\ & \vdots \\ {\left(x-x_{m}\right)}^{2} & +{\left(y-y_{m}\right)}^{2}=z_{m}{}^{2} \end{array}$$

where, the *z_m_* means the distance from the target to the *m*th WiFi detector estimated by PPRM. From the over-determinant equations above, we can find that the greater the number of WiFi detectors is, the higher the positioning accuracy is. Equation (17) can be solved by many methods, such as least squares method (LSM) [43]. Although the LSM-based positioning algorithm might minimize the sum of mean-squared error, it cannot guarantee that any estimated point is optimal, which lead to a series of high-error positioning ones [37]. To deal with this issue, this paper employs the Taylor series expansion to estimate the target location. Nevertheless, to ensure the convergence of the algorithm and timeliness, Taylor series expansion needs to estimate the initial target location, which cannot deviate too far from the actual position. Therefore, the initial position obtained by LSM is taken as the input of Taylor series expansion in this study.

Assume the initial position in the iteration is (*x*′, *y*′), and let corresponding distance from the initial position to the WiFi detector are *d*_1_′, *d*_2_′, …, *d_m_*′, respectively. The actual coordinate can be expressed as the summation of the coordinates obtained by LSM and the position offset:

(18) $$\left\{ \begin{array}{l} x=x^{\prime }+\Delta x\\ y=y^{\prime }+\Delta y \end{array}\right.$$

Meanwhile, the first order Taylor series expansion is imported to reduce computational complexity as follows:

(19) $$f(x,y)=f(x^{\prime }+△x,y^{\prime }+△y)=f(x^{\prime },y^{\prime })+\frac{\partial f(x,y)}{\partial x}|x^{\prime }\Delta x+\frac{\partial f(x,y)}{\partial y}|y^{\prime }\Delta y$$

Consequently, we can obtain the following equation:

(20) U=YΔ

where $Y={\left[\begin{array}{l} \frac{x^{\prime }-x_{1}}{d_{1}^{\prime }},\frac{x^{\prime }-x_{2}}{d_{2}^{\prime }},\ldots ,\frac{x^{\prime }-x_{n}}{d_{n}^{\prime }}\\ \frac{y^{\prime }-y_{1}}{d_{1}^{\prime }},\frac{y^{\prime }-y_{2}}{d_{2}^{\prime }},\ldots ,\frac{y^{\prime }-y_{n}}{d_{n}^{\prime }} \end{array}\right]}^{T}$, $U={\left[d_{1}-d_{1}^{\prime },d_{2}-d_{2}^{\prime },\ldots ,d_{n}-d_{n}^{\prime }\right]}^{T}$, $\Delta ={\left[\Delta x,\Delta y\right]}^{T}$.

The solution of Equation (20) can be formulated by:

(21) $$\Delta ={\left(Y^{T}Y\right)}^{-1}Y^{T}U$$

Each iteration needs to determine whether |Δ*x*| + | Δ*y*| is less than the threshold *δ* (set to 0.01 [37]) or not.

#### 3.3.3. Positioning Optimization Based on Unscented Kalman Filter (UKF)

Based on the aforementioned methods, we can obtain the estimated location coordinate of each moving target at each moment *t*. In fact, in the urban road environment, the movement of pedestrian is continuous, and the position at time *t* has a strong correlation with the previous interval *t*−1. In [32,33,34], the filter techniques have been used to improve the localization performance when the accuracy is unsatisfied. Basically, they applied the previous localization coordinates as model inputs to restrict the possible move trace to reduce the error. In [32], the authors demonstrated that UKF is superior to the Kalman filter (KF) and extended Kalman filter (EKF) in solving moving target-tracking problems. Therefore, this study uses UKF to smooth the estimated points obtained by the LS-TSE. In the UKF, it is necessary to carefully define the noise covariance matrix *Q* and measurement noise covariance matrix *R,* and the covariance matrix *G* before performing prediction and updating steps. In this study, the initial values of matrices *Q*, *R* and *G* are the same as in [32]. Finally, the output of UKF ${\hat{x}}_{t},{\hat{y}}_{t}$ is regarded as the ultimate positioning coordinate.

## 4. Experiment Results

### 4.1. Experiment Description

**Figure 3:**
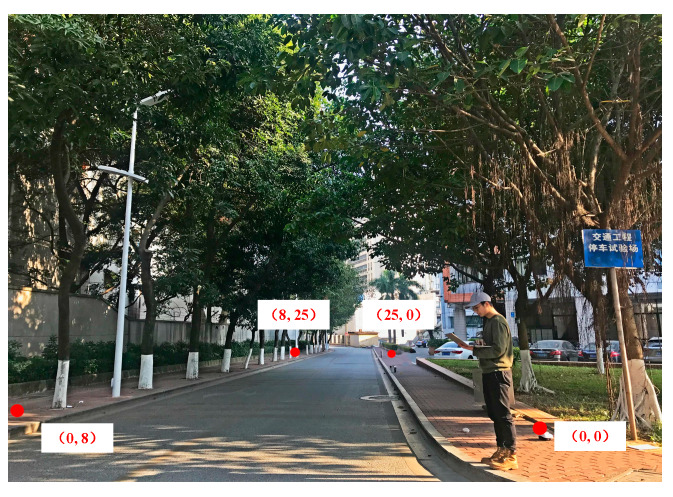
Experiment environment.

To evaluate the performance and application of the proposed localization scheme, we carried out field experiments on 23 January 2019 at the Wushan campus, South China University of China, Guangzhou. The site is located in Zhujiangnan Rd around Jiaotong Building. Figure 3 plots the floor layout, where a 2-D coordinate system is used to describe the coordinates of each point, and the origin one is chosen as the right-bottom corner point of the road. As shown in Figure 3, two high buildings (Zhonggongjiaoyu building of 27 floors on the left, and Jiaotong building of 6 floors on the right) line both sides of the road, and other obstacles are around the site, such as cars, pedestrians, bicycles, trees, trucks, metal bars, etc.

Integrated WiFi detectors of DS-007 manufactured by Chengdu DataSky Company of China, have been proved to be suitable for outdoor environments [40]. The field experiments in our testbed showed the effective coverage area of a DS-007 detector can be approximated as a sphere with a radius of 30 m or large. In our experiments, we deployed four WiFi detectors, an android terminal as user device (Galaxy Note 2 manufactured by Samsung, Seoul, Korea) and a notebook computer as the data collection server (Xiaoxin Air 13 manufactured by Lenovo, Beijing, China). According to the width and length of the target area, four WiFi detectors were deployed at the same height on both sides of the road and their coordinates were set to (0, 0), (25, 0), (0, 8), (8, 25) in meters, respectively. Also, the scanner data (MAC address, RSSI, and timestamp) collected by WiFi detectors were real-time updated into data server machine by one second.

At the offline stage, we conducted field experiments on the 25 m-length road link from 0 to 25 m by 1 m steps, where 200 replicated experiments at each tested point were repeated such as to reduce the random error. Therefore, the total size of data samples is 25 × 200 = 5000, which includes the calibrated target position and corresponding WiFi scanner data including the target’s MAC, timestamp and RSSI value. Among the data, 70% was selected as a training set to calibrate the RSSI-distance relationship model, and the remainder was used for fitting performance validation. At the online stage, the tester held the smartphone with the constant speed as much as possible during walking. Several reference points with surveyed locations were used to generate the ground truth, and a stopwatch was used to record the time when the tester passes these reference points. Then, the locations of the ground truth between two adjacent reference points are generated through interpolation while assuming that the tester walks at a constant speed. To eliminate human body’s impact on the experimental results, we unified the way of holding smart phone in the experiments.

### 4.2. Physical Distance Estimation Evaluation via Single Detector

The performance of PPRM-based distance estimation is discussed in this section. In the estimation, the selected range for polynomial degree is set to *n* = 2, 3,4, 5, and the corresponding PPRM models are called 2-polynomial (POLY2), 3-polynomial (POLY3), 4-polynomial (POLY4), and 5-polynomial (POLY5), respectively. We compare our proposed algorithms with the traditional PM and PRM.

#### 4.2.1. RSSI-Distance Formula Based on PPRM

**Figure 4:**
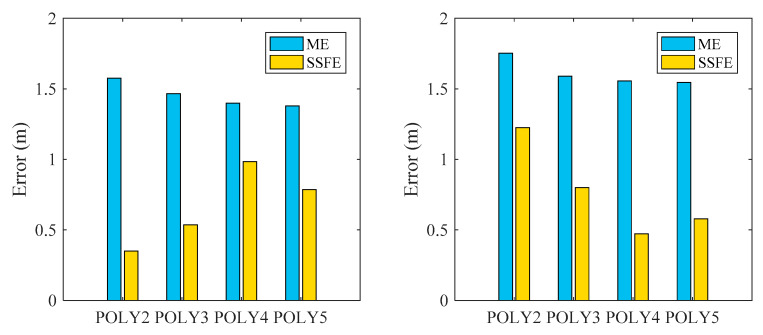
Mean error (ME) and sum of the square fluctuation errors (SSFE) of the *n*th-degree polynomial: (**left**) first fitting result; (**right**) second fitting result.

In the first-stage fitting, the ME and SSFE of the *n*th-degree polynomial were calculated for the training set, and the result is shown in Figure 4(left). One can find that the ME of POLY2, POLY3, POLY4 and POLY5 are 1.58, 1.47, 1.40, 1.38, respectively, while the difference is no more than 0.2. Meanwhile, the SSFE of four fitting functions are 0.35, 0.54, 0.98 and 0.78, respectively, whereas the difference is up to 0.63. In order to minimize the fluctuation error of the fitting function, the polynomial with the least SSFE is chosen as the optimal fitting function. Therefore, POLY2 is chosen for the first fitting function. This result is consistent with the result of Zhuang et al. [11], who found that POLY2 is the optimal fitting function with better performance and the least computation load.

However, we also find the RSSI distribution will dynamically change when the physical distance exceeds a certain value. Also, the individual estimated error and the mean error in POLY2 are calculated according to Equations (10) and (11) as shown in Table 1. For example, the ME is about 1.58 m, while the errors of three estimated points at 15, 14 and 13 m are 3.58, 2.70 and 2.08 m, respectively. According to the previous criterion in Equation (12), the errors of three consecutive estimated points are greater than the mean, and the piecewise polynomial fitting should be set at the 15 m point. This is consistent with the findings of the study [37], which further proves that when the transmission distance exceeds a certain threshold, the attenuation speed of RSSI strength will change.

In the second-fitting phase, the ME and SSFE of the *n*th-degree polynomial are recalculated based on the corresponding training dataset as shown in Figure 4(right). The SSFE values of POLY2, POLY3, POLY4 and POLY5 are 1.23, 0.80, 0.47 and 0.58, respectively, while the difference is up to 0.76. Therefore, POLY4 is chosen for the second fitting function. This may be caused by the fluctuation of RSSIcollected by far-side sensors being much larger than near-side ones. In other words, if these errors are not corrected, the positioning accuracy will worsen. Table 2 summarizes the individual and mean error of PLOY4. It is obvious that the first fitting function from 0 to 15 m does not need to be split because only two or less consecutive estimated points (8 and 9 m) has the errors greater than the mean in terms of Equation (12). Therefore, the RSSI-distance relationship is divided into two segmentations in this study. The first segmentation is 0–15 m, and the second is 16–25 m.

Therefore, the final RSSI-distance estimation can be formulated as follows:

(22) $$d=\left\{ \begin{array}{l} 0.00071\times s^{4}+0.19118\times s^{3}+19.22231\times s^{2}+854.76607\times s+14173.091,s>-78.57\\ 0.02297\times s^{2}+2.6\times s+73.84,s<-78.57 \end{array}\right.$$

where, *s* is the measured value of the RSSI. Notably, at the offline phase, *s* denotes the RSSI value via Gaussian filtering; at the online phase, *s* denotes the real-time RSSI value via CVKF filtering.

#### 4.2.2. Physical Distance Estimation at the Static Points

**Figure 5:**
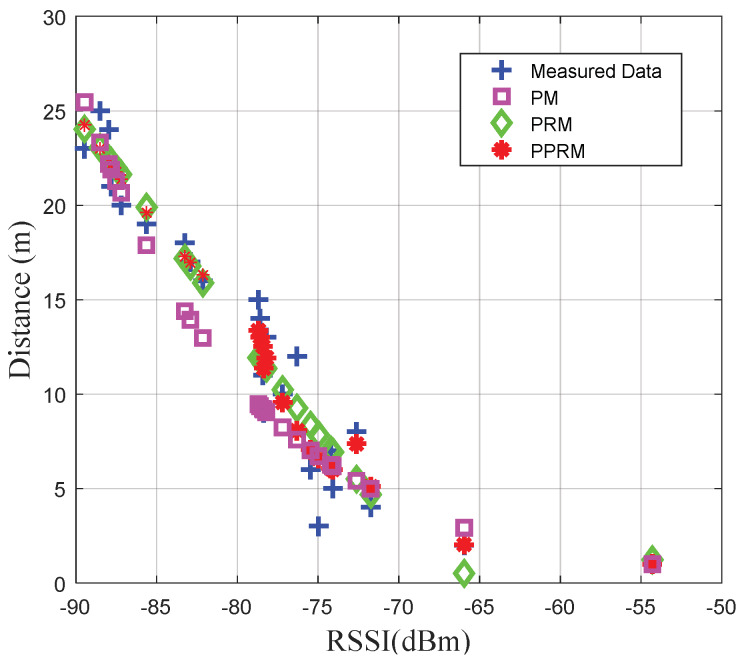
The offline fitting results between received signal strength indicator (RSSI) and distance under three models.

**Figure 6:**
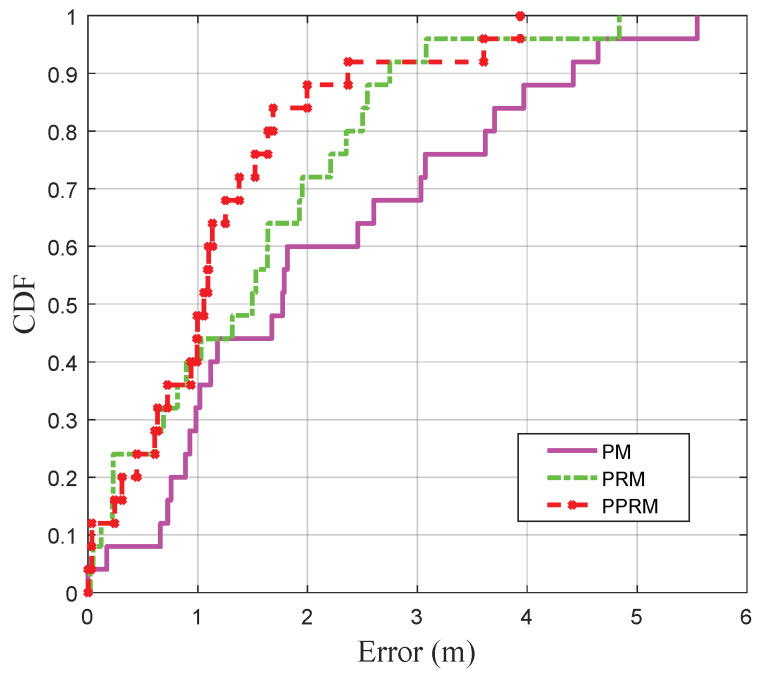
Cumulative distribution functions (CDFs) of offline distance estimation errors under three models.

In order to validate the performance of PPRM in the target stationary environment, three methods of PM, PRM, and PPRM are tested to fit the RSSI-distance in the urban road environment, respectively. The coefficients of PM are already corrected, and the fitting degree of PRM is set to *n* = 2 [11]. Figure 5 shows that the proposed PPRM outperforms PM and PRM. It seems that the relationship between RSSI and distance does not obey the log path-loss model due to the effect of attenuation, reflection, multipath, etc. Meanwhile, Figure 6 depicts the cumulative distribution functions (CDFs) of the distance estimation error obtained by PM, PRM and PPRM, respectively. The 90 percentiles of distance estimation error by PPRM is not greater than 2.48 m, which increases to 4.46 m by PM and 2.79 m by PRM, respectively. Therefore, our tests indicate that PPRM can provide much higher accurate and reliable distance estimation than the popular PM and PRM in the urban road environment.

### 4.3. Pedestrian Real-Time Positioning Evaluation via Multi-Detector

#### 4.3.1. Analysis of Physical Distance Estimation from Real-Time Data

In practice, the signal propagation environment is always complex and diverse, which may result in high fluctuation of collected RSSI data. The ability to tolerate RSSI fluctuations is one of the important performance indexes for an RSSI-based localization system. In our previous research work [40], we proved that the CVKF algorithm is a better filter for smoothing the great RSSI fluctuation in the outdoor environment. In order to further improve the estimation accuracy, a combination of CVKF and PPRM is tested to fit the RSSI-distance with the comparison of PM, PRM, and PPRM. In the field experiment, the tester held the mobile terminal and walked straight from the reference point (0, 0) to the reference point (25, 0), and then returned to the origin (0, 0).

**Figure 7:**
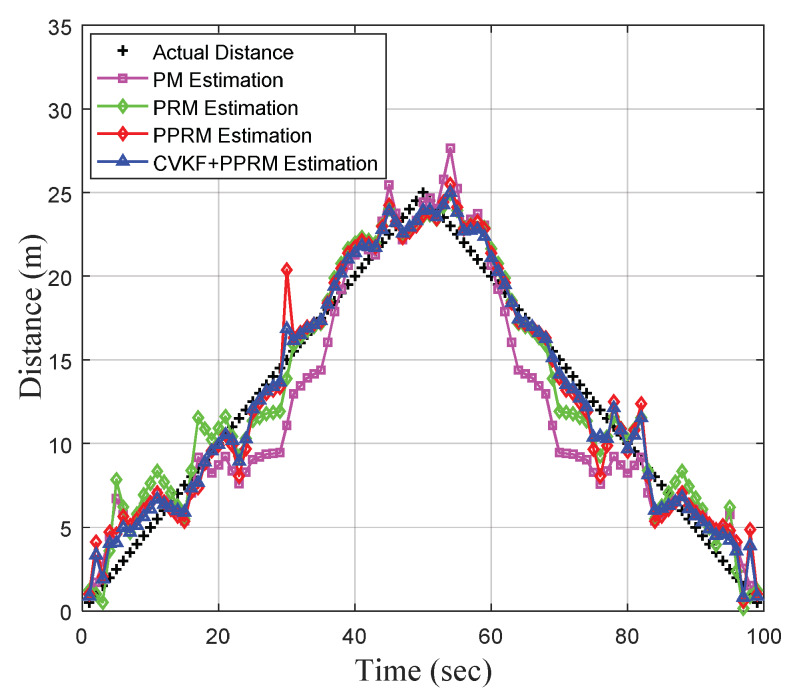
Real-time physical distance estimation under four models.

**Figure 8:**
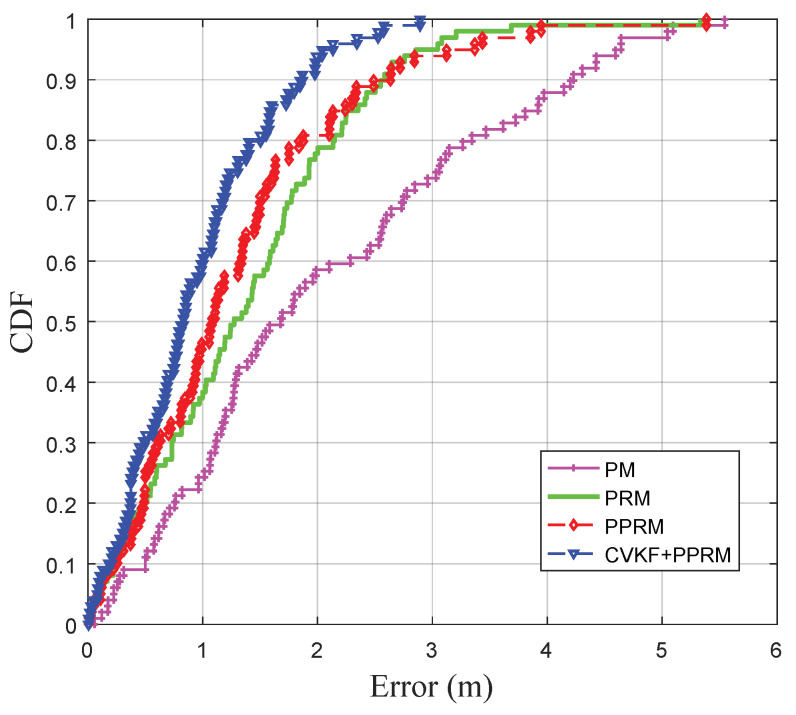
CDFs of real-time distance estimation errors under four models.

Figure 7 shows the measured data and estimated distance, and Figure 8 descripts the CDFs of the distance estimation errors by PM, PRM, PPRM and CVKF+PPRM. The results show that the proposed CVKF+PPRM outperforms other three models in real-time distance estimation while the target keeps moving. Notably, average error obtained by CVKF+PPRM is only 0.93 m, which outperforms the existing PM (2.05 m), PRM (1.37 m), and PPRM (1.27 m). In Figure 8, the 90 percentiles of distance estimation error by CVKF+PPRM is not greater than 1.98 m, which increases to 4.24 m by PM, 2.84 m by PRM, and 2.76 m by PPRM, respectively. To sum up, one can find that the PPRM fitting model after data filtering by CVKF shows promise in improving dynamic estimation accuracy.

#### 4.3.2. Analysis of Target Positioning Estimation Results via Multi-Detector

In the previous section, we have demonstrated how to estimate the target distance to a specific WiFi detector, and proved that the CVKF+PPRM framework can handle the non-linear estimation problem associated with the RSSI-distance. This section will discuss the localization performance based on LS-TSE fusion via multi-detector. The average error (AE) and root mean square error (RMSE) are imported to evaluate the proposed algorithms, which represent the closeness between the target’s estimated position ${\hat{x}}_{t},{\hat{y}}_{t}$ and the actual one (*x_t_*, *y_t_*) at a specific time *t*. In detail, the corresponding measurement of effectiveness (MOE) indexes are defined as follows [32].

Average localization error (error in *X*-*Y* estimates):

(23) $$ALE=\frac{1}{T}\sum_{t=1}^{T}\sqrt{{\left({\hat{x}}_{t}-x_{t}\right)}^{2}+{\left({\hat{y}}_{t}-y_{t}\right)}^{2}}$$

Root mean squared error (RMSE):

(24) $$RMSE_{X}=\sqrt{\sum_{t=1}^{T}\frac{{\left({\hat{x}}_{t}-x_{t}\right)}^{2}}{T}}$$

(25) $$RMSE_{Y}=\sqrt{\sum_{t=1}^{T}\frac{{\left({\hat{y}}_{t}-y_{t}\right)}^{2}}{T}}$$

(26) $$RMSE_{avg}=\frac{\left(RMSE_{X}+RMSE_{Y}\right)}{2}$$

**Figure 9:**
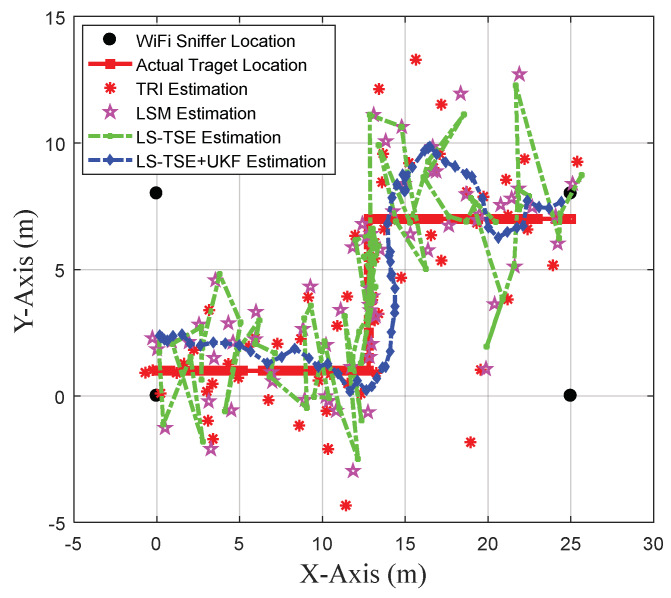
Target trajectories estimated by trilateral localization (TRI), least squares model (LSM), least squares Taylor series expansion (LS-TSE) and LS-TSE+unscented Kalman filter (UKF).

Compared with LS-TSE, trilateral localization (TRI) [40] and the least squares model (LSM) [43], the combined LS-TSE+UKF can outperform them as shown in Figure 9. The TRI-based algorithm selects the RSSI data collected by the WiFi detectors located at three reference points of (0, 0), (25, 0), (0, 8), and the UKF parameter is determined based on the literature [30]. The tester straightly keeps walking from the origin point (0, 1) to end (25, 7) via the intermediate ones (13, 1) and (13, 7), and Figure 9 illustrates the collected actual and estimated target trajectories in *X*-*Y* coordinates under different models of LS-TSE+UKF, LS-TSE, LSM and TRI.

**Figure 10:**
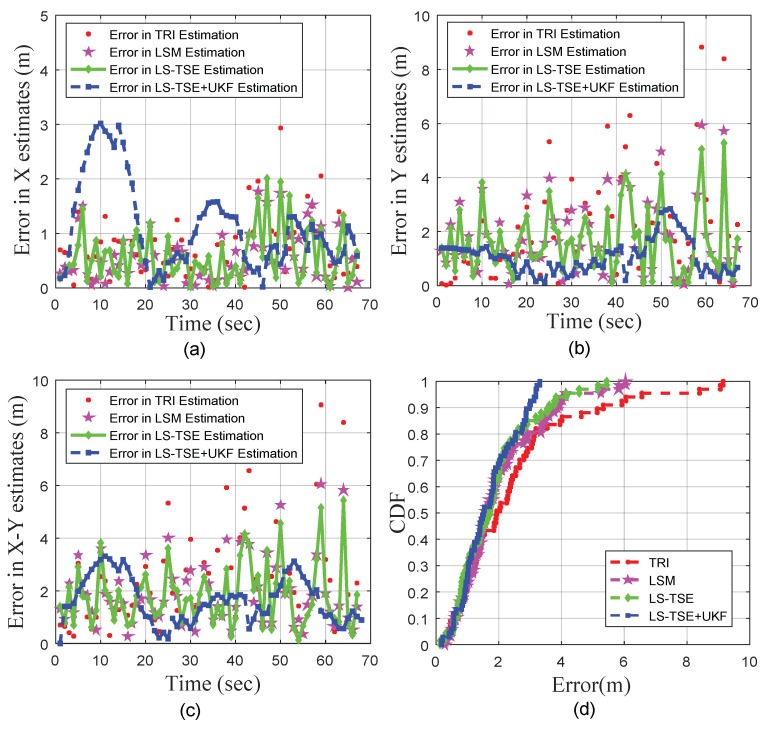
Comparison of Localization errors: (**a**) X axis; (**b**) Y axis; (**c**) X-Y axis; (**d**) CDFs of 2-dimensional location errors.

Meanwhile, the Figure 10a–c demonstrates the comparisons of localization errors on *X*-axis and *Y*-axis under four mentioned algorithms. In the experiment, the target moves along the *X*-axis during the initial 30 s. The average positioning error of the proposed LS-TSE+UKF algorithm on the *X*-axis is only 1.14 m. However, it is worth noting that the LS-TSE+UKF has a peak error between 5 s to 15 s, which might be due to the fact that the UKF has a time-lag and sensitivity to initial values. Then, the LS-TSE+UKF begins to converge after 15 s, and the positioning error on the *X*-axis gradually drops. Meanwhile, the average positioning error of LS-TSE+UKF on the *Y*-axis is only 1.06 m, which outperforms the existing TRI algorithm (2.14 m), LSM algorithm (1.77 m), and LS-TSE algorithm (1.64 m).

Table 3 also shows the minimal, maximal and average localization error as well as the RMSE in *X*, *Y* and *X*-*Y* axis. Figure 10d and Table 3 reveal that our proposed algorithm has the ability to closely follow the actual target’s trajectory. The average localization error of the LS-TSE+UKF is 1.67 m, which is outperforms the existing TRI algorithm (2.44 m), LSM algorithm (1.96 m), and LS-TSE algorithm (1.82 m). Moreover, the average RMSE of LS-TSE+UKF drops approximately 33.67%, 10.81%, and 5.04% compared to that of TRI, LSM, and LS-TSE, respectively. Meanwhile, the CDFs of the localization errors shows the proposed combination of LS-TSE and UKF has a higher reliability than others. In details, one can find that the LS-TSE+UKF localization scheme could achieve error of <2.99 m with the probability of 90% or more, which increases to 5.33 m by TRI, 3.85 m by LSM, and 3.62 m by LS-TSE, respectively. In summary, it can be easily concluded that the proposed LS-TSE + UKF outperforms other algorithms in the urban road environment.

### 4.4. Complexity Discussion

Finally, we compare the average computing time of the four algorithms implemented in Matlab on a computer (processor 2.2 GHz Intel Core i5-8500, Memory 8 GB and Windows 10 operation system). The average computing time of TRI, LSM, LS-TSE and LS-TSE + UKF are 0.286, 0.347, 2.685 and 3.214 s, respectively. It is observed that the proposed approach has a reasonable complexity compared to other approaches. Given the prominent improvement of LS-TSE+UKF localization accuracy over other methods, such moderately increased complexity may be acceptable.

## 5. Conclusions

In this paper, we have proposed a new range-based localization method based on the integration of the piecewise polynomial regression model (PPRM), constant velocity Kalman filter (CVKF), least squares Taylor series expansion (LS-TSE) and unscented Kalman filter (UKF) for low-speed pedestrian positioning by using WiFi scanner data. Firstly, the proposed method uses CVKF to filter the real-time RSSI value and estimate the straight-line distance between the target and WiFi detector via PPRM. Then, a filtered algorithm with UKF is developed to smooth the subset of estimated 2-dimentional positioning points, which can be obtained by LS-TSE. Finally, the UKF output points are regarded as the ultimate positioning solution. The proposed model uses the trajectory-based fitting technique considering the kinetic continuity of a pedestrian, which is capable of reducing peak errors and can achieve high positioning accuracy for moving traffic pedestrians.

Field experiment results show that the combined CVKF and PPRM can achieve highly accurate Euclidean distance estimation having the average error of 1.98 m with the probability of 90% at the offline fitting stage, which outperforms the existing propagation model (PM) (4.24 m), polynomial regression model (PRM) (2.84 m), and PPRM (2.76 m). Meanwhile, for 2-dimensional localization of single low-speed moving target at the online phase, it can achieve the average positioning error of 1.67 m which is much better than trilateral localization (TRI) (2.44 m), the least squares method (LSM) (1.96 m), and LS-TSE (1.82 m), respectively. Furthermore, our proposed localization method can be easily realized in the practical application and can promote the development of a robust WiFi-based positioning scheme.

In this study, the test environment is a typical urban road scenario with only a single pedestrian moving. In our future work, we will extend the experiments on different urban sidewalks with masses of pedestrians for the validation of the reliability and effectiveness of the proposed localization method under various geometric configurations.

## Figures and Tables

**Table 1:** The errors between each point and the fitting point after the first fitting in meters.

Actual Distance	Fitting Distance	Error	Actual Distance	Fitting Distance	Error
25	23.57	1.43	12	9.12	2.88
24	22.78	1.22	11	11.14	0.14
23	24.98	1.98	10	9.94	0.06
22	22.13	0.13	9	11.09	2.09
21	22.57	1.57	8	6.10	1.90
20	21.68	1.68	7	7.32	0.32
19	19.54	0.54	6	8.38	2.38
18	16.52	1.48	5	7.23	2.23
17	16.09	0.91	4	5.47	1.47
16	15.17	0.83	3	7.95	4.95
15	11.42	3.58	2	2.23	0.23
14	11.30	2.70	1	0.35	0.65
13	10.92	2.08	Mean Error	1.58	

**Table 2:** The errors between each point and the fitting point after the second fitting in meters.

Actual Distance	Fitting Distance	Error	Actual Distance	Fitting Distance	Error
15	13.36	1.64	7	6.06	0.94
14	13.01	0.99.	6	7.06	1.06
13	11.91	1.09	5	6.00	1.00
12	8.06	3.84	4	5.10	1.10
11	12.52	1.52	3	6.60	3.60
10	9.56	0.44	2	2.03	0.03
9	12.37	3.37	1	1.00	0.01
8	5.37	2.63	Mean Error	1.56	

**Table 3:** The errors between each point and the fitting point after the second fitting in meter.

Positioning Algorithm	Min Error	Max Error	Average Error	RMSE in *X*-axis	RMSE in *Y*-axis	Average RMSE in *X*-*Y*
TRI ^1^	0.28	9.15	2.44	0.97	3.00	1.99
LSM ^2^	0.27	6.04	1.96	0.73	2.23	1.48
LS-TSE ^3^	0.13	5.44	1.82	0.74	2.03	1.39
LS-TSE+UKF ^4^	0.18	3.32	1.67	1.40	1.24	1.32

^1^ Trilateral localization (TRI); ^2^ Least squares model (LSM); ^3^ Least squares Taylor series expansion (LS-TSE); ^4^ LS-TSE+unscented Kalman filter (LS-TSE+UKF).
